# Lectures on the Diagnosis and Treatment of Diseases of the Lungs

**Published:** 1841-02-06

**Authors:** W. W. Gerhard


MEDICAL EXAMINER.

DEVOTED TO MEDICINE, SURGERY, AND THE COLLATERAL SCIENCES.

No. 6.] PHILADELPHIA, SATURDAY, FEBRUARY 6, 1841. [Vol. IV.

### LECTURES ON THE DIAGNOSIS AND TREATMENT OF DISEASES OF THE LUNGS.

BY W. W. GERHARD, M. D.

### LECTURE XIII.—(*Continued.*)

#### HECTIC FEVER.

*Hectic Fever* is a very frequent consequence of tubercles after they have attained a certain stage of development,—not that the fever is peculiar to tubercles, but, on the contrary, it is common to all diseases attended with suppurating cavities communicating with the exterior. It scarcely occurs under other circumstances,—that is, the true hectic; the fever of irritation, on the contrary, is very frequent, when no suppuration exists, and is then very analogous to the initiatory fever of ordinary tuberculous disease. The true hectic occurs in the advanced stages of phthisis, when softening of tuberculous matter has taken place, and a pus-secreting cavity is formed. It is characterized, as is well known, by a strong tendency to a regular paroxysmal type, which sometimes approaches closely to intermittent, and by a pulse, which is at least as frequent, but generally more compressible than that of the early irritative fever.

We may add to the general symptoms of phthisis the extreme exhaustion and tendency to œdema which occur in the latter stages of the disease. These, of course, are not peculiar to it.

2. *Symptoms directly dependent upon the development of tubercles in the lungs.* The bronchial or other inflammations which occur very early in phthisis, are not properly dependent upon this disorder if they precede it, but the true secondary inflammation of the lungs is a necessary consequence of the tuberculous deposit, and is strictly consecutive to it. The signs of the inflammation are of course scarcely different from those of ordinary bronchitis, and have been sufficiently noticed already,—that is, if we restrict the term bronchitis to those cases in which the inflammation extends over a large surface, and is in itself tolerably severe; but if the slighter cases of bronchial irritation, in which a cough occurs very early in connection with tubercles, are to be regarded as instances of bronchitis, the symptoms are very different from those of ordinary catarrh. It is not possible to discriminate between the influence of the slight bronchial inflammation and of tubercles in the production of the cough. We therefore class both these causes together, and regard the cough which occurs at the commencement of phthisis as the result of either; this is at first very insignificant, and sometimes, though rarely, quite absent. At first it is much more frequent early in the morning than at any other period of the day, although you will find a great irregularity in this respect; it gradually increases in severity, and in the frequency of its return, until at last it becomes severe and more or less paroxysmal. This occurs when cavities of some size have formed, and the liquid contained in them tends gradually to accumulate until it gives rise to a violent paroxysm of cough. In the last stages of the disease the cough becomes feeble and hollow, or cavernous in its character; a circumstance which is familiar to every one who has seen many cases of consumption.

The expectoration is of course nearly connected with the cough; at first it is, like the cough, very slight, and often insignificant; but, after a time, it becomes more and more abundant, and of the usual bronchitic character, for there is either no purulent matter, or this is so small in quantity as not to attract notice. After the tubercles have begun to soften, pus is necessarily found in the sputa, and those are of a yellowish colour, differing often in appearance from ordinary muco-purulent sputa, for the softened tuberculous matter of which they are in great part composed, is extremely viscid and different in appearance from pure pus. If the softening is very rapid, the quantity of the thick pasty substance often amounts to ten or twelve ounces in twenty-four hours. In general, it is combined with more or less thin mucus, which is intermixed with the thick yellow matter. As soon as cavities form, the thicker, more purulent part of the sputa, which is retained in the cavities, is moulded into a rounded, irregular form, often with loose, cottony edges; these portions are suspended, if they contain air, or if not, they fall to the bottom of the transparent mucus. This constitutes the nummular sputa, which are not characteristic of phthisis in general, but only of one stage of it. If the cavities become hard, and cease entirely, or in great part, to secrete purulent matter, the expectoration consists merely of thin mucus, as the lining membrane does not materially differ from that of the bronchial tubes. In the advanced stages of phthisis, and occasionally at a rather earlier period when the strength of the patient is much enfeebled, the walls of the cavity may soften down rapidly, and fall into a fœtid, thick, grayish liquid; this is nothing else than gangrene of a tissue partly filled with tuberculous matter.

The gradual obstruction of the lung with tuberculous matter, and its removal by softening, renders so large a portion of the vesicles unfitted for purposes of respiration, that the dyspnœa is always considerable in the advanced stages of phthisis. In the earlier period, however, this will ofteu occur to a greater or less extent, so that dyspnœa is very far from being a mere mechanical result of the obstruction, but is in part caused by the vital action going on in the lungs. It is most severe in acute phthisis.

There is almost no pain from tubercles, properly so called; the uneasiness felt from time to time in the chest seems to depend entirely upon the accompanying inflammation. The local signs purely belonging to phthisis, with the exception of the cough and expectoration, are slight; those belonging to the secondary inflammations are very numerous; even the cough and expectoration may be nearly absent, owing to causes which, in many cases, are not understood. We know, however, that the same causes which render other pectoral diseases latent, act here,—that is, the feebleness of the patient, and the diseased condition of the brain. Hence in lunatics we find that phthisis is always obscure, and sometimes scarcely betrayed by any local symptoms.

*Physical signs.*—These are amongst the most decided in advanced cases, but very obscure in the early periods of the disorder. We do not now refer to the signs of the concomitant inflammations, but to those of phthisis, properly so called. At first these are limited to the signs of mere obstruction; the vesicular inspiration is feeble or harsh, and slightly puerile, while the expiration is becoming louder and louder. The character of the respiration, therefore, gradually becomes rude, and at last approaches the bronchial, in which it terminates as soon as the vesicular structure is completely replaced by the tuberculous matter. The bronchial respiration is more or less local, according to the quantity of tubercle, and the more or less obstruction of the larger bronchi themselves: if these remain uncompressed, the air of course passes freely through them, and the bronchial respiration may be tolerably loud; if, on the other hand, they are soon closed, the respiratory sounds are all feeble, as soon as softening begins, a slight rhonchus is heard, approaching more nearly to the sub-crepitant than any other, this gradually passes into decided crackling, and finally into gurgling, as the liquid becomes more abundant, and the cavity increases in size. The cavernous respiration is generally developed with the gurgling, and sometimes replaces or alternates with it.

The signs of percussion are of course limited in phthisis to those of induration of the parenchyma; they give us no information as to the progress or approach of softening. As the tubercles are generally most developed at the summit of the lungs, the dulness is first perceptible there; hence it may often be first detected by percussing above the clavicle, or upon, or immediately beneath it; and however slight the dulness may be, there is little difficulty in distinguishing it, if attention be paid to the natural degree of resonance, and to the comparison of the two sides. The intercurrent inflammation may, of course, give rise to varying degrees of dulness, which may rapidly increase or diminish.

The signs of percussion and auscultation are the most important, but in the course of the disease, attention should be paid to the conformation of the thorax. The parietes of course contract when pleuretic adhesions have taken place; even if there are no adhesions, the consolidation of the lung produces a partial contraction of the tissue, which causes a slight sinking of the ribs; the most important, however, is caused by the adhesions. These are most perceptible near the clavicles and behind them. The same causes render the ribs comparatively motionless in the same place, as the air enters imperfectly into the tissue which is thus hardened.

It naturally occurs to every one that these signs are rather applicable to the advanced than to the early stages of the disorder ; but there are generally some characters which afford a tolerably good indication of commencing phthisis, as soon as a slight deposit has formed, or a partial infiltration of the tissue has taken place. These are not so much signs which are referrible to any of the fixed classes which I described at the early period of this course, but mere trivial alterations of the natural respiratory sounds, which become important from their position and the coincidence of the general symptoms of common phthisis ; without these, the signs are of some value, though a very limited one. Thus the commencement of a rude respiration, which is denoted by a trifling increase of expiratory sound, especially if in the left side, or a harsh rough inspiratory murmur, which differs from the natural vesicular sound, are both of some value, if they are combined with a slight dulness on an extremely careful percussion. That is, always with the proviso, that the general symptoms should be in some degree developed, for I cannot repeat too often that the general signs are at the commencement of the disease the most important. But if the physical signs are added, a probable opinion may be converted into a certain one, which affords a good measure of the degree of the disorder; if they are absent, the importance of the general signs is diminished, but not destroyed.

The physical signs of deposit and softening of tubercle extend gradually over the lungs, in proportion to the progress of the disorder, until a considerable portion may be involved. But the parts last affected do not offer as well marked characters as those first attacked ; hence the respiration in the parts which remain comparatively healthy, becomes in a great degree supplementary and puerile ; and, even when tubercles have invaded it, the vesicles still dilate, and their peculiar murmur is still loud and harsh, notwithstanding a certain number of them may have become impervious to the air.

3. *Symptoms dependant upon the accessary disease of the lungs and air passages, including larynx and trachea.*—To a great extent the remarks relative to these affections have been already anticipated, from their necessary connection with the subjects previously treated of. Thus the secondary inflammation of the bronchi produces few symptoms differing from those of the tuberculous disease of the lungs ; the bronchitis, however, may occasionally become acute, and thus the rapid increase of the cough and dyspnœa, and the formation of the characteristic rhonchi, establish the nature of the intercurrent affection. The sputa are also often increased in quantity, and become more transparent, like those of the earlier stages of ordinary bronchitis. Pneumonia too gives rise to increased dyspnœa, and to more or less crepitus and fullness of respiration, with frequently a viscid transparent expectoration ; but the bronchial respiration is much less loud than in ordinary pneumonia, and the increase of dyspnœa is much less considerable than we might *a priori* suppose it to be. In other words, the chronic disease modifies the symptoms of the acute affection. The secondary pleurisy is almost always of the dry kind. Effusion sometimes, however, takes place during more advanced stages of tuberculous disease, but this is rather an exception than a rule ; the ordinary symptoms of the pleurisy are pains which vary from a mere stitch to a severe, sharp, lancinating pain, preventing the patient from lying on the affected side. The flying or wandering pains which are at times felt in the thorax during the course of phthisis, are probably dependent upon the same pleuritic complication, although this is not perfectly certain.

The inflammation of the larynx and trachea has a much more important connexion with phthisis. Chronic laryngitis is often called laryngeal phthisis, which is a sufficient proof that a close connexion or a great similarity was supposed to exist between these diseases. When the affection of the larynx occurs late in phthisis, it is absolutely secondary, and results, in part at least, from the irritation of the sputa passing from the lungs over the larynx and trachea, and thus giving rise to inflammation and ulceration ; but the form of chronic laryngitis which attracts most attention is that in which the lesion has preceded the disease of the lungs, and for a long time may appear totally independent of it. But after a time, which is very variable as to length, the signs of consolidation of the lungs are apt to supervene, and the case may then terminate in decided phthisis. From our knowledge of this frequent connexion, we must be cautious as to the prognosis of such cases. It is true that if the laryngitis can be arrested at a tolerably early stage, the patient will probably not become consumptive; but should it resist our efforts of cure, the disease almost always terminates in a tuberculous affection: this is the case both with the common and syphilitic varieties of acute laryngitis. Of course the existence of a highly developed tuberculous diathesis greatly enhances the danger of the case, and, under these circumstances, the laryngitis is sometimes little else than the commencement of the morbid phenomena.

The same remarks apply to chronic trachitis, except that it is a more obscure affection, not connected with a special function like the larynx. The symptoms are generally merely cough, with an obscure sensation of tickling or soreness above and immediately below the upper margin of the sternum; while those of laryngitis, in addition to the sensation of irritation, are hoarseness, gradually passing into aphonia. The trachitis is less important in itself than the laryngitis, unless there be some evidence of general tuberculous disorder, when it is quite as grave. Like the laryngitis, it should be removed as soon as possible.

The disease known by the name of chronic pharyngitis, or sometimes "clergyman's sore throat," is occasionally connected with phthisis. But the connection is rather an accidental than a fixed one, for the disorder consists essentially in an inflammation of the fauces, including uvula and tonsils. It is certainly rather more apt to occur in individuals who offer the characters of the scrofulous diathesis, than in others; and it has apparently some agency in favouring the development of phthisis in these individuals.

